# Supplementary material for: Health system and quality of care factors contributing to maternal deaths in East Java, Indonesia
Source: PLoS One. 2021 Feb 26;16(2):e0247911. doi: 10.1371/journal.pone.0247911 (PMC7909698; doi:10.1371/journal.pone.0247911)
Supplement: Attachment — Submitted filename: Response to Reviewers - PONE-D-20-09737.docx [file pone.0247911.s001.docx]

**Ref: PONE-D-20-09737**

**Health System and Quality of Care Factors Contributing to 522 Maternal Deaths in East Java, Indonesia**

We are thankful to the reviewers for their detailed review and very helpful suggestions. We have revised the paper. The changes made with reference to each of the points raised by the reviewers appear below. Please note that the line numbers in the ‘track change’ copy and the ‘clean’ copy manuscript match when the ‘track change’ document is viewed in ‘Simple Markup’ view (MS Word). However, as most of the text pertaining to the basic review of 522 deaths was removed, the line numbers do not match when the document is viewed in ‘All Markup’ view.

**REVIEWER #1:**

This paper presents an extraordinary overview of the systemic processes that have resulted in an unacceptable level of maternal mortality in East Java. All too often we are lured into a false sense of security by the ongoing introduction of advancing technologies that serve to improve clinical outcomes, not recognizing that the salutary impact of that process is the proper implementation of those technologies and, perhaps most importantly, the development of a clinical system that accurately evaluates an individual and provides for the proper clinical setting and staff that can best utilize technology and expertise to assure optimal outcomes. This paper clearly describes a situation where, despite the availability of appropriate clinical technology, a broken system serves to maintain an inappropriate rate of maternal mortality. The paper is easy to follow but suffers slightly from occasional poor style and syntax, as well as an occasional spelling error, scattered throughout the manuscript. Nonetheless, this paper presents critically important information to our readers and to obstetrical providers and administrators worldwide.

We are thankful for the commendation of the work. We are committed to and continue working with the local departments and hospitals for improved quality of care and better health outcomes for pregnant women and babies.

To improve the style, syntax, grammar and spelling, a native English speaker Associate Prof John Moss, a co-author, has edited the paper.

**REVIEWER 2:**

Overall, the paper is very long. I do not think there is substantial additional value from the details of the 522 deaths, or the 70 deaths. The information in these sections has been widely demonstrated in previous published literature. I suggest removing these sections, and concentrating on telling a much more concise story about the deaths that were analysed in detail. The introduction and the discussion should also be substantially reduced.

We are thankful for the detailed review and thoughtful suggestions. We agree that this basic information (review of 522 provincial deaths and 70 hospital deaths) may not add substantial value and is already widely demonstrated in the literature. We have removed those sections. That information was provided to define the context for the in-depth analysis of 30 deaths. In the revised draft, we have summarised that contextual information in the **Setting** section and provided summary information on 70 deaths in the **Results** section. To provide a context, we have kept summary information about the provincial and hospital deaths (**Lines 124-127 & 151-159**).

**Minor points**:

The manuscript would benefit from review by a native English speaker to correct minor errors e.g. in abstract and introduction “now receiving antenatal care for an adequate number of times”

A native English speaker, Associate Professor John Moss has reviewed and edited the revised paper for style, syntax, grammar and spelling.

Line 68 “In East Java in 2016, 97% and 78% of women had at least one and four ANC visits (respectively) to skilled attendants”. Please describe the proportion of women who have a skilled attendant at delivery.

We have added the information, “95% of women had a skilled attendant at delivery (3)”. (**Line 67**)

Line 70: “Recently, the East Java MMR has stagnated with little reduction.” Please reference this data.

Reference #2 to the East Java Health profile and the MMR over the last few years has been inserted.

Line 72 Please reconsider this statement. The paper by Hogan shows a steadily decreasing MMR everywhere except sub Saharan Africa, specifically demonstrated in Web figure 6c.

We have revised this as suggested. The revised statement reads, “Over the last few years, the East Java MMR has stagnated with little reduction (2). Yet internationally there is a steadily decreasing MMR everywhere except in sub Saharan Africa (4)”. Reference 4 is Hogan. (**Line 70**)

Line 82 “Better performing health systems and good quality care are needed to reduce these deaths, the majority of which now occur at the time of labour and delivery and in the postpartum period (Renfrew 2014). “ This sentence appears to simply repeat the previous sentence and could be removed.

The sentence has been deleted.

Line 123 – line 142. I am unclear if this section refers to the current paper, or the previous paper by the same author. If it refers to the previous paper, I do not think it needs to be included with this degree of detail.

The method described is for this research. We have provided further details on selection criteria, numbers and analysis in the **Method** section.

Table 1 – this is a very detailed table that doesn’t provide much additional information. An attempt should be made to describe the uncertainty, or variability around these numbers from year to year. Comparisons regrading rates of death by age or parity, as has been done throughout the manuscript are not very useful without knowing the age/parity of the entire population of women giving birth.

***This table has been removed***, as part of removal of the information on the 522 deaths and the tightening of that on the 70 deaths. We agree with the reviewer’s suggestion to remove the passage, considering that this basic information did not add substantial value and is already demonstrated in the literature. We have removed these sections. That information was provided as a context. In the revised version, we have summarised that contextual information in the **Setting** section and provided a summary of information on the 70 deaths in the **Results** section.

269: concomitant illness. This list varies from very mild disease to very severe disease which was very likely to contribute to maternal death (left to right shunt and Eisenmengers disease).

We have added a statement that, “While these concomitant illnesses contributed to the complexity and severity, not all concomitant illnesses were the main cause of death. They varied from mild diseases to very severe disease.” (**lines 291-193**)

276: This caesarean section rate seems consistent with the very high caesarean rate for the hospital, as described in line 156, and so does not support the argument that the women were in critical condition.

This is a peak referral hospital where most of the women are transferred from frontline hospitals when they have a condition or complication that cannot be managed at the latter. We have revised the statement to read, “As almost all of the pregnant women at this hospital are transferred from other hospitals, they tend to be suffering from obstetric complications and many are in a critical condition. Overall, of the 1,370 women who were delivered at this hospital in 2017, 793 were delivered by C-Section” (**line 200**).
